# Decreased Left Putamen and Thalamus Volume Correlates with Delusions in First-Episode Schizophrenia Patients

**DOI:** 10.3389/fpsyt.2017.00245

**Published:** 2017-11-20

**Authors:** Xiaojun Huang, Weidan Pu, Xinmin Li, Andrew J. Greenshaw, Serdar M. Dursun, Zhimin Xue, Haihong Liu, Zhening Liu

**Affiliations:** ^1^Mental Health Institute of the Second Xiangya Hospital, Central South University, Changsha, China; ^2^The China National Clinical Research Center for Mental Health Disorders, National Technology Institute of Psychiatry, Key Laboratory of Psychiatry and Mental Health of Hunan Province, Changsha, China; ^3^Medical Psychological Institute, Second Xiangya Hospital, Central South University, Changsha, China; ^4^Department of Psychiatry, University of Alberta, Edmonton, AB, Canada; ^5^Mental Health Center of Xiangya Hospital, Central South University, Changsha, China

**Keywords:** delusions, first-episode schizophrenia, voxel-based morphometry, putamen, thalamus

## Abstract

**Background:**

Delusional thinking is one of the hallmark symptoms of schizophrenia. However, the underlying neural substrate for delusions in schizophrenia remains unknown. In an attempt to further our understanding of the neural basis of delusions, we explored gray matter deficits and their clinical associations in first-episode schizophrenia patients with and without delusions.

**Methods:**

Twenty-four first-episode schizophrenia patients with delusions and 18 without delusions as well as 26 healthy controls (HC) underwent clinical assessment and whole-brain structural imaging which were acquired a 3.0 T scanner. Voxel-based morphometry was used to explore inter-group differences in gray matter volume using analysis of covariance, and Spearman correlation coefficients (rho) between the Scale for the Assessment of Positive Symptoms (SAPS)-delusion scores and mean regional brain volumes was obtained.

**Results:**

Patients with delusions showed decreased brain gray matter volumes in the left putamen, thalamus, and caudate regions compared with HC. Patients with delusions also showed decreased regional volume in the left putamen and thalamus compared with patients without delusions. SAPS-delusion scores were negatively correlated with the gray matter volumes of the left putamen and thalamus.

**Discussion:**

Left putamen and thalamus volume loss may be biological correlates of delusions in schizophrenia.

## Introduction

In the Diagnostic and Statistical Manual of Mental Disorders (5th ed.; DSM-5), delusion is described as a false belief based on inferences about external reality that is firmly sustained in the mind despite the inconvertible and obvious proof to the contrary (1). As one of the most prominent symptoms of schizophrenia, delusion carries significant clinical value. There are many types of delusions in schizophrenia, including delusion of control, delusion of jealousy, delusion of reference, delusion of thought insertion and thought broadcasting, persecutory delusion, religious delusion, delusion of guilt, and somatic delusions (2). However, little is known about the neurobiological underpinnings of delusion (3). Identifying the neural substrate of delusion would provide an important insight into the mechanism of schizophrenia, a highly heterogeneous disease (4).

Results from previous investigations into the structural substrates of delusions in schizophrenia have been controversial, and several brain areas have been implicated (5–10). For example, negative correlations were reported for severity of delusional symptoms in relation to gray matter volume in the dorsomedial frontal cortex, left claustrum, and right insula (7, 8), but positive correlation has been reported in relation to gray matter volume in the superior temporal gyrus (5, 6). Somatic delusions are found to be associated with reduction in left fronto-insular gray matter volume (9). Involvement of subcortical structures, such as the thalamus and the basal ganglia, was observed when delusion was evaluated together with other positive symptoms of schizophrenia, such as hallucinations and thought disorders (10). Moreover, a structural study has provided evidence of the association of delusions of reference with decreased gray matter density in the caudate nucleus in early psychosis (11). Researchers proposed that delusions may arise from defective error monitoring, which is a neural process thought to be located in the anterior cingulate/dorsomedial frontal cortex (12–14). Despite these observations, the definitive neuronal substrates of delusion remain elusive. In this context, interpretation of research findings is complicated by heterogeneity in symptoms of schizophrenia, along with variations in the medication, treatment methodology and the lack of reliable animal models that can mimic behavior related to delusional experience (15). An obvious and promising strategy is examination of whole-brain differences between schizophrenic patients with and without delusions while controlling for other symptoms and medications. Conceivably, patients with delusions may show specific changes in gray matter volume. This strategy, with voxel-based morphometry (VBM), has been used in studies of bipolar disorder patients, yielding correlations of brain structural abnormalities with psychopathological symptoms (16). Clearly, a study focusing on patients diagnosed with schizophrenia, with and without delusions, and exploring the structural differences with a whole-brain strategy may be helpful in furthering our understanding of delusions in this context.

In this study, therefore, we performed structural magnetic resonance imaging (MRI) on patients diagnosed with schizophrenia, with and without experience of delusions, in comparison with matched healthy controls (HC). Furthermore, to minimize the impacts of illness duration and medication on patients, this study focused on a unique sample of schizophrenic patients who were experiencing their first episode and were drug naïve or having used antipsychotics for less than 8 weeks prior to scanning. We hypothesized that patients experiencing delusions would show decreased regional gray matter volumes compared with patients without delusions and HC, respectively, and that the magnitude of these volume differences would correlate with delusion severity.

## Materials and Methods

### Participants

Forty-three first-episode patients, diagnosed with schizophrenia, were recruited from the inpatient and outpatient units of the Second Xiangya Hospital, Central South University, Changsha, China. One participant withdrew due to phobia of MRI machines. The patients were assessed using the Structured Clinical Interview for DSM-IV Axis-I Disorders, Patient Edition (SCID-I/P) (17). The diagnosis and symptom ratings of schizophrenia were made independently by two psychiatrists.

Patients met the following inclusion criteria: (1) 18–45 years of age; (2) Han Chinese ethnicity; (3) completed nine or more years of education; (4) right-handed as determined by the Annett Hand Preference Questionnaire (18); and (5) meeting the DSM-IV diagnostic criteria for schizophrenia. Participants were excluded if they had (1) a history of neurological disorder or other serious physical illness; (2) a history of severe medical disorder; (3) a history of substance abuse as reported by participants and confirmed with collateral sources, such as medical records and close relatives; (4) a contraindication to MRI; or (5) a history of electroconvulsive therapy.

All patients were in their first-episode of diagnosis for schizophrenia, with illness duration of less than 18 months. To minimize the effects of antipsychotic medication, only drug-naïve patients and those having used antipsychotics for less than 8 weeks were recruited, details of medications in drug-treated patients are provided in Section “Results.”

Twenty-six HC were recruited from the community in the Changsha City area. Except for not meeting the DSM-IV criteria for any Axis-I psychiatric disorders, or having a family history of psychiatric illness among their first-degree relatives, inclusion and exclusion criteria were equivalent to the patients group.

After the risks and benefits of the study were discussed with each potential recruit into this study, written informed consent was obtained from every participant. The study was approved by the ethics committee of the Second Xiangya Hospital of Central South University.

### Symptom Assessments

For all patients diagnosed with schizophrenia, positive and negative symptoms were assessed using the Scale for the Assessment of Positive Symptoms (SAPS) (19, 20) and the Scale for the Assessment of Negative Symptoms (SANS) (21), respectively. The assignment of the patients to the group with or without delusions was based on the delusions subscale scores from the SAPS. Global Rating of Severity of Delusions is rated from 0 (None) to 5 (Severe) and the score of 2 (delusion definitely present but at times the patient questions the belief) means mild existence of delusion (19, 20). The patients with a score of 2 or higher were assigned to the group with delusions (SCHd) and those with delusion score of less than 2 were assigned in the group without delusions (SCHnd).

### Image Acquisition and Processing

Three-dimensional T1-weighted images were acquired sagittally on a 3.0-T Philips Achieva whole-body MRI scanner (Philips, The Netherlands) using turbo field echo sequence with the following parameters: repetition time = 7.5 ms, echo time = 3.7 ms, flip angle = 8°, field of view = 240 mm × 240 mm, acquisition matrix = 256 × 200, slice thickness = 1 mm, gap = 0, number of slices = 180. All subjects were instructed to move as little as possible, and foam pads were used to minimize head motion.

T1 images were processed with VBM version 8 based on the Statistical Parametric Mapping 8 package (SPM8; Wellcome Trust Centre for Neuroimaging, London, UK; http://www.fil.ion.ucl.ac.uk/spm) and Matlab 7.10 (The Mathworks, Inc.). The T1 images were segmented by one expert (Weidan Pu) into gray matter, white matter, and cerebrospinal fluid (CSF) using VBM8 software. Default parameters were used, including Gaussian intensity distributions per class: 2 for gray matter/white matter/CSF, 3 for bone, 4 for other soft tissues, and 2 for air; bias regularization: 0.0001; cutoff for bias FWHM: 60 mm; affine regularization to ICBM space template (average-sized template); warping regularization: 4; and sampling distance: 3. We used High-dimensional Diffeomorphic Anatomical Registration using Exponentiated Lie Algebra for spatial normalization (22). A spatial adaptive nonlocal means denoising filter was applied to the data. We also used a Markov Random Field weighting factor of 0.15 and light cleanup of partitions. Nonlinear modulated normalized gray matter images were generated. This enables analysis of relative difference in regional gray matter with correction for individual brain size. The images were displayed and reviewed to ensure that segmentation and normalization procedures worked properly. For checking quality of the image data, the sample homogeneity of these resulted images was also tested by using covariance approach based on VBM8. All images were spatially normalized to the T1-weighted template in the Montreal Neurological Institute space and were resampled into a final voxel size of 1.5 mm × 1.5 mm × 1.5 mm. Finally, we smoothed the normalized gray matter images modulated for nonlinear components using a Gaussian kernel of FWHM 8 mm in each direction. The subsequent voxel-wise statistical comparison of regional gray matter between groups was obtained from the smoothed gray matter images.

### Statistical Analysis

Statistical analyses of participant demographic and clinical characteristics were carried out using the Statistical Package for the Social Sciences (SPSS 19.0 for Windows). Comparisons of demographic variables were made using analysis of variance or independent-samples *t*-tests for continuous variables and chi-square tests for dichotomous variables.

Since age, sex, and antipsychotic medication have been reported to impact brain structure and functions (23, 24), we included these variables in our analysis of covariance (ANCOVA) in SPM8. An overall *F*-test was performed, followed by *post hoc t*-tests on significant regions from the *F*-test analysis. Voxel-based *t*-tests were performed for gray matter atrophy in regions with a significant *F*-test, comparing SCHd versus HC, SCHnd versus HC, and SCHd versus SCHnd. The significance level was set at *p* < 0.05 [false discovery rate (FDR) corrected] with a minimum cluster size of 50 voxels (1 voxel = 1.5 mm × 1.5 mm × 1.5 mm). The brain regions identified from the comparative analysis were extracted as a mask for calculating the mean gray matter volume for each subject. The Spearman correlation coefficient (rho) between delusion severity score and the mean volume of each region was calculated by using SPSS 19.0 for Windows, and Bonferroni corrections were used to adjust for multiple comparison. In addition, we were interested in whether antipsychotic medications and duration of illness were related to gray matter loss in the putamen and thalamus in patients, so we determined correlation coefficients between gray matter volume and chlorpromazine-equivalent dosage (CPZ), and between gray matter volume and the duration of antipsychotic treatment.

## Results

### Demographic and Clinical Characteristics

The demographics profiles, clinical characteristics, and diagnoses as confirmed by two psychiatrists with an inter-rater reliability of *k* ≥ 0.80 are shown in Table 1. No significant differences were observed among the three participant groups for age, gender, or years of education. The patients with delusions (SCHd) had higher SAPS total scores and SAPS delusion scores than those without delusions (SCHnd). SCHnd had higher SANS anhedonia scores than SCHd. There were, however, no inter-group differences on the other SAPS or SANS subscales, or in duration of illness, duration of treatment, or CPZ dosage.

**Table 1:** Demographic and clinical characteristics.

Variable	HC (*n* = 26)	SCHnd (*n* = 18)	SCHd (*n* = 24)	*F*/*t*/χ^2^	*p*-Value
	Mean (SD)	Mean (SD)	Mean (SD)		
Age (years)	23.15 (5.36)	22.50 (4.46)	24.25 (6.64)	0.53	0.59
Gender (male/female)	17/9	12/6	14/10	0.39	0.82
Education (years)	12.92 (1.72)	12.47 (2.59)	12.46 (2.35)	0.35	0.71
SAPS total	–	8.39 (5.94)	19.38 (6.76)	5.49	<0.00*
SAPS hallucinations	–	0.50 (1.04)	0.67 (0.96)	0.54	0.60
SAPS delusions	–	0.17 (0.38)	3.17 (1.05)	12.90	<0.00*
SAPS bizarre behavior	–	0.61 (0.92)	1.04 (1.12)	1.33	0.19
SAPS positive formal thought disorder	–	0.39 (0.70)	0.79 (1.02)	1.44	0.16
SANS total	–	41.50 (23.54)	26.79 (24.91)	−1.94	0.06
SANS affective blunting	–	1.94 (1.43)	1.25 (1.39)	−1.58	0.12
SANS alogia	–	1.78 (1.31)	1.08 (1.32)	−1.70	0.10
SANS avolition/apathy	–	2.28 (1.53)	1.54 (1.38)	−1.63	0.11
SANS anhedonia	–	2.89 (1.13)	1.75 (1.36)	−2.88	0.01*
SANS inappropriate attention	–	1.22 (1.31)	1.17 (1.09)	−0.15	0.88
Illness duration (months)	–	8.42 (4.49)	9.28 (5.23)	0.56	0.58
Chlorpromazine equivalents (mg)	–	252.78 (210.37)	237.92 (232.04)	−0.22	0.83
Duration of treatment (days)	–	15.72 (17.13)	8.25 (5.52)	−1.78	0.09

*HC, healthy controls; SCHnd, patients diagnosed with schizophrenia without delusions; SCHd, patients diagnosed with schizophrenia with delusions; SAPS, Scale for the Assessment of Positive Symptoms; SANS, Scale for the Assessment of Negative Symptoms*.

*p < 0.05.

Among the 42 schizophrenia patients, all but one, who was drug naïve, was receiving antipsychotic treatment, with duration of treatment of less than 8 weeks. Among drug-treated patients, 37 received drugs during scanning period and four experienced a washout period of more than 2 weeks before recruitment. Of the patients receiving antipsychotics, 37 were receiving atypical antipsychotics (clozapine, risperidone, quetiapine, olanzapine, aripiprazole, and ziprasidone), one was on a typical antipsychotic (sulpiride), and three were receiving combined typical and atypical antipsychotics (two of them receiving olanzapine and sulpiride, and another one receiving risperidone and sulpiride).

### Inter-Group Differences in Gray Matter Volume

**Figure 1:**
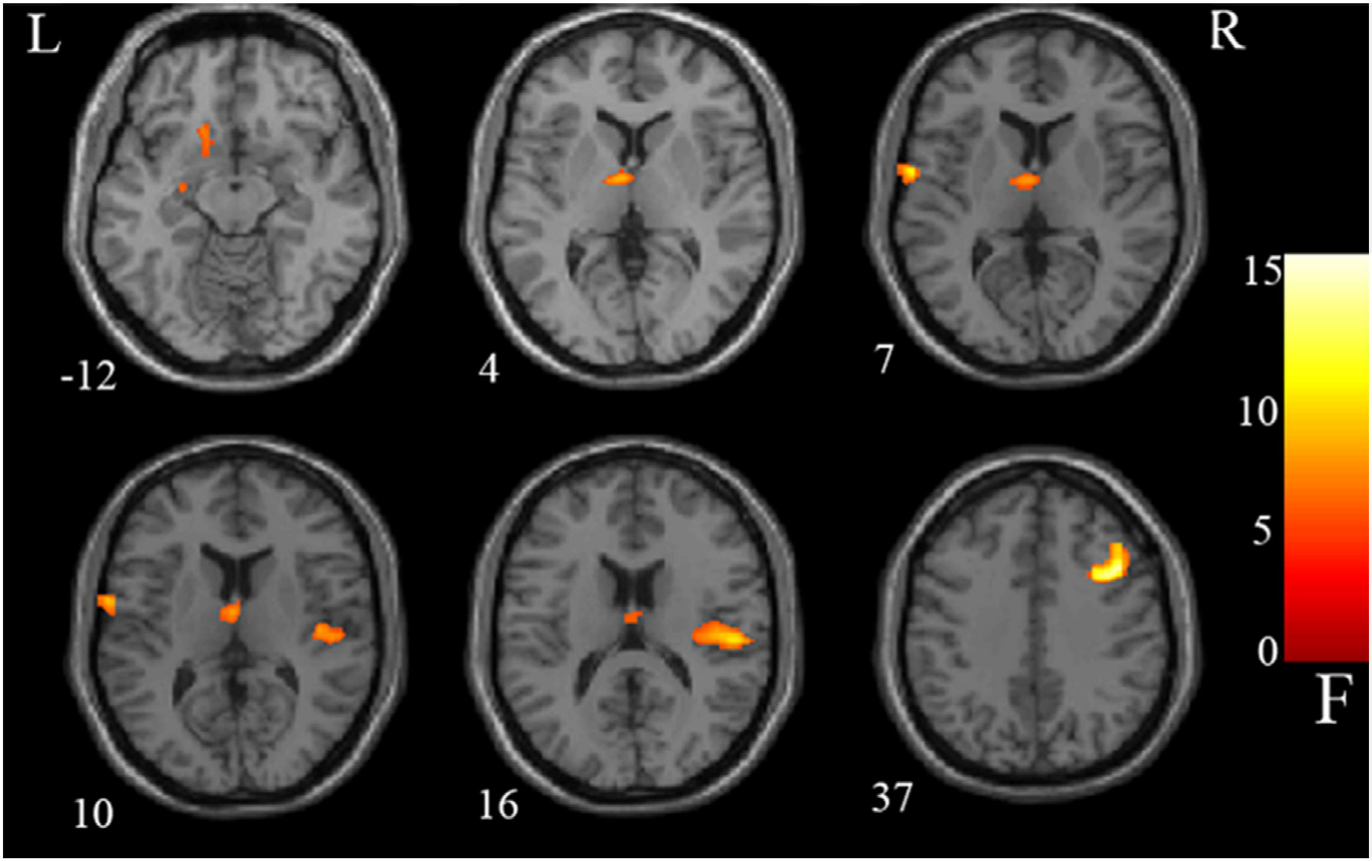
Group difference: analysis of covariance results. Gray matter differences between three groups were detected in the left putamen, left caudate, left thalamus, left Rolandic operculum, right Heschl, right Rolandic operculum, and right middle frontal gyrus. Statistical inferences were made with a voxel-level statistical threshold (*p* < 0.05) after false discovery rate correction for multiple comparisons. The color bar represents the range of *F* values.

The VBM analysis showed that gray matter abnormalities were significantly different among the three groups in the left putamen, left caudate, left thalamus, left Rolandic operculum, right gyrus of Heschl, right Rolandic operculum, and right middle frontal gyrus (ANCOVA, FDR Corrected, *p* < 0.05; Figure 1).

**Figure 2:**
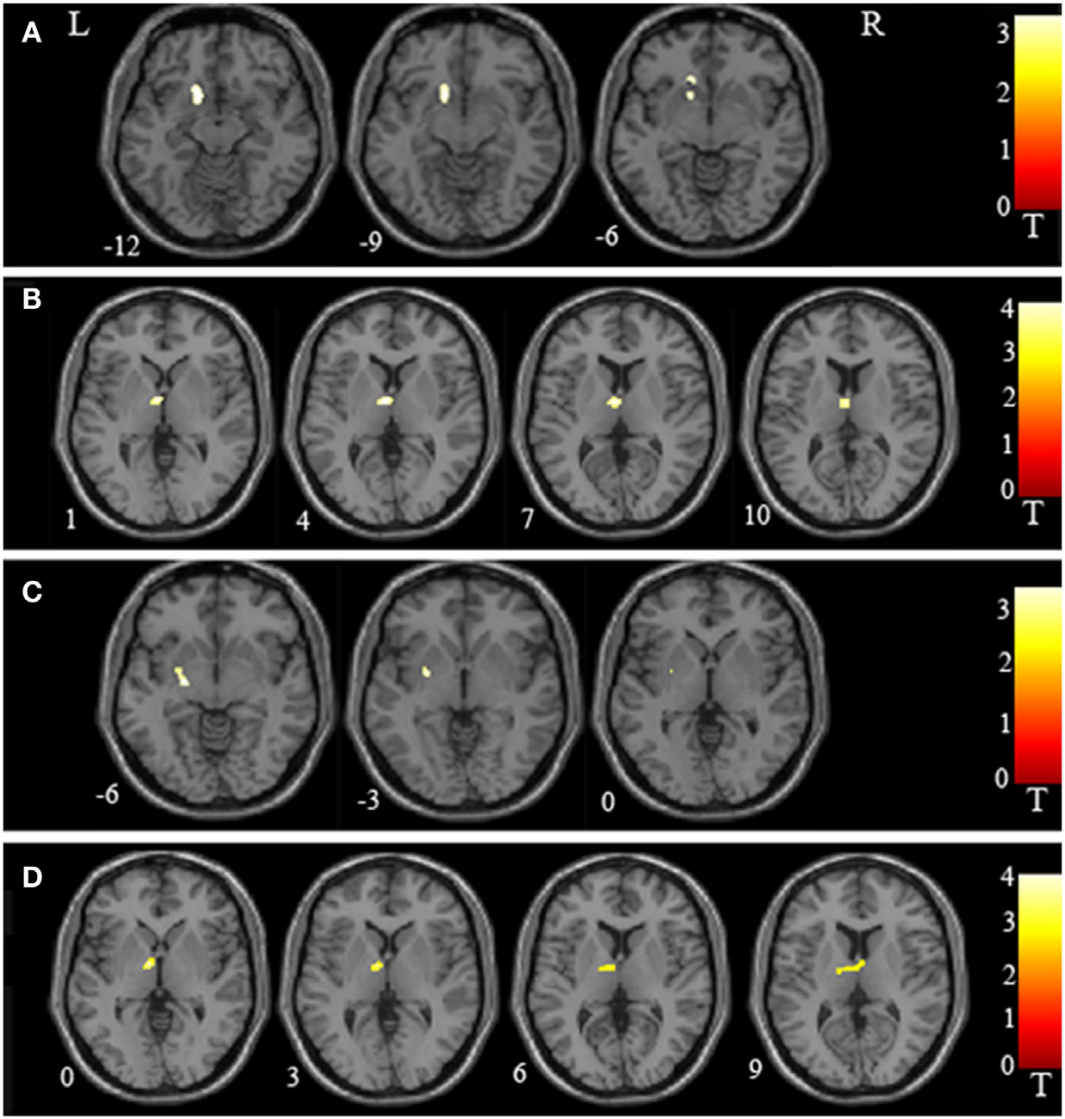
Regional volume abnormalities in the putamen and thalamus as identified in inter-group voxel-based morphometry. **(A)** Schizophrenia patients with delusions (SCHd) had lower regional volume in the left putamen than healthy controls (HC). **(B)** SCHd had lower regional volume in the left thalamus than HC. **(C)** SCHd had lower regional volume in the left putamen than schizophrenia patients without delusions (SCHnd). **(D)** SCHd had lower regional volume in the left thalamus than SCHnd. L, left; R, right. The color bars represent the range of *t* values.

*Post hoc t*-test results in Table 2 and Figure 2 (*p* < 0.05, FDR corrected) demonstrate that the SCHd group showed significant decreases in regional gray matter volume in the left putamen, thalamus, and caudate compared with the HC group. The SCHd group also had decreased volume in the left putamen and thalamus compared with the SCHnd group. In addition, both SCHd and SCHnd showed increased volume in the right middle frontal gyrus relative to HC. SCHnd had decreased volumes in the bilateral Rolandic operculum and the right Heschl gyrus relative to HC. SCHnd had less right Rolandic operculum volume than SCHd.

**Table 2:** Inter-group differences of gray matter volume (*p* < 0.05, FDR corrected).

Brain region	Cluster size (voxels)	MNI coordinates (*X*, *Y*, *Z*)	Peak *t* value
**HC > SCHd**			
L. thalamus	92	−5, −6, 4	4.06
L. putamen	61	−14, 14, −12	3.71
L. caudate	92	−10, 13, −12	3.71
**HC < SCHd**			
R. middle frontal gyrus	496	42, 18, 34	5.46
**HC > SCHnd**			
L. Rolandic operculum	141	−62, −1, 7	5.02
R. Rolandic operculum	161	51, −21, 13	3.89
R. Heschl	102	45, −16, 10	3.5
**HC < SCHnd**			
R. middle frontal gyrus	354	42, 15, 37	6.03
**SCHnd > SCHd**			
L. putamen	59	−26, −3, −3	3.06
L. thalamus	147	−14, −9, 9	3.06
**SCHnd < SCHd**			
R. Rolandic operculum	359	53, −22, 16	4.33

*HC, healthy controls; SCHnd, patients diagnosed with schizophrenia without delusions; SCHd, patients diagnosed with schizophrenia patients with delusions; L, left; R, right; MNI, Montreal Neurological Institute*.

### Correlation Analysis

**Figure 3:**
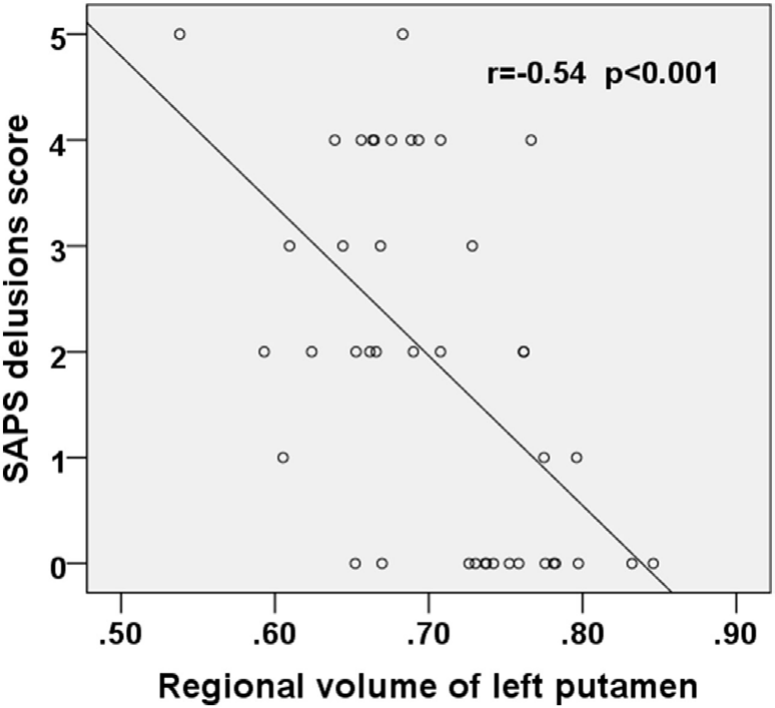
Scatter plot showing negative correlation between the SAPS delusions score and the regional volume of the left putamen in the first-episode schizophrenia patients. SAPS, Scale for the Assessment of Positive Symptoms.

**Figure 4:**
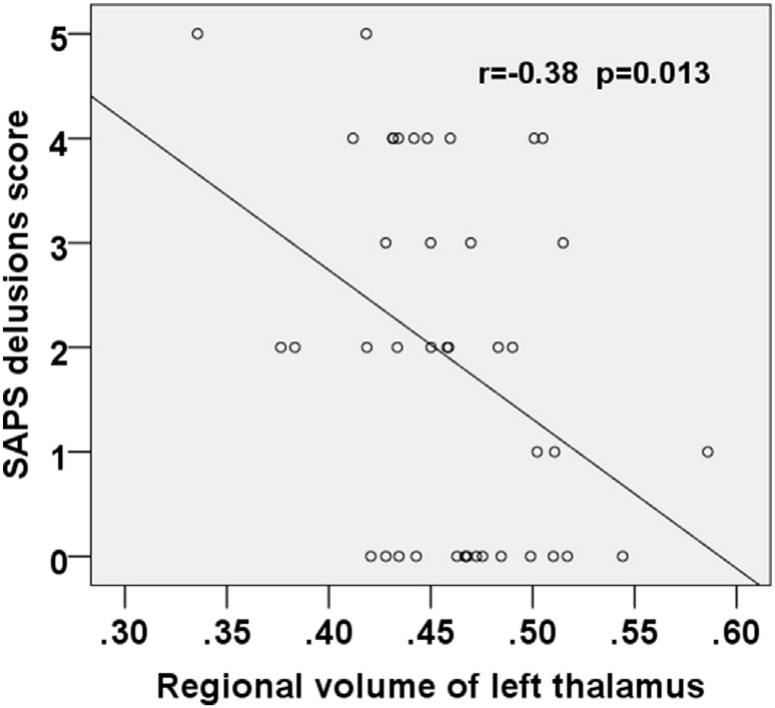
Scatter plot showing negative correlation between the SAPS delusions score and the regional volume of the left thalamus in the first-episode schizophrenia patients. SAPS, Scale for the Assessment of Positive Symptoms.

The SAPS delusion scores correlated negatively with gray matter volume of the left putamen (rho = −0.54, *p* < 0.001) (Figure 3) and thalamus (rho = −0.38, *p* = 0.013) (Figure 4); these results remained significant after Bonferroni correction for multiple comparison (*p* < 0.025, Table 3). No correlations were observed between regional gray matter volumes and CPZ-equivalent medication dosage or duration of treatment (*p* > 0.05).

**Table 3:** Spearman’s correlation coefficients between the regional gray matter volume and clinical measures in the first-episode schizophrenia (*n* = 42).

Regions	SAPS delusions score		CPZ (mg)		Duration of treatment (days)	
	*r*	*p*	*r*	*p*	*r*	*p*
L. thalamus	−0.38	0.01*	−0.26	0.10	−0.09	0.56
*L. putamen*	−0.54	0.00*	0.07	0.66	−0.23	0.15

*SAPS, Scale for the Assessment of Positive Symptoms; CPZ, chlorpromazine equivalents; L, left; R, right*.

*p < 0.025 (Bonferroni correction for multiple comparison).

## Discussion

Using a VBM strategy, we found that patients with delusions had decreased gray matter volume in the left putamen and thalamus relative to patients without delusions and HC. Except for SANS-anhedonia and SAPS-delusion scores, there were no inter-group differences in any other SANS or SAPS subscale scores between patients with and without delusions. The SAPS-delusion score correlated negatively with gray matter volume of the left putamen and thalamus, which suggests that these may be key structures for the biological underpinning of delusions.

In this study, we observed left putamen and thalamus had decreased gray matter volumes in patients that were also correlated with delusion scores. This is consistent with another study that identified psychotic symptoms in a patient with schizophrenia as resulting from disruption in the left putamen and thalamus (25). Furthermore, another prior study demonstrated that alteration of fronto-limbic activity, related to the striatal novelty processing, was associated with the delusions (26). Conversely, Zhu et al. (27) suggested that patients diagnosed with schizophrenia that exhibited severe delusions have relatively normal structural integrity compared with patients without delusions. Two possible explanations for these seemingly contradictory results are related to differences in the groups of patient diagnosed with schizophrenia. First, in this study, there are no inter-group differences in any other SAPS or SANS subscales except anhedonia and delusion scores, while these subscale scores were not addressed in the other study (27). Second, all patients in this study were in their first-episode of schizophrenia, either completely drug naïve or having used antipsychotics for less than 8 weeks. Kitabayashi et al. (28), in accord with our results, reported the emergence of psychotic symptoms in a patient with a right putamen abnormality, and Devinsky (29) reported a complex relationship in which delusions may result from right hemisphere lesions leading to left hemisphere activity-based delusions. In this study, the author argued that the left hemisphere released from right hemispheric influence domination over gestalt and emotional familiarity would be overbiased related to categorization, resulting in delusional thinking. However, other studies have suggested that a lack of frontal and right hemispheric inhibition, respectively, unleashes a “creative narrator” from the monitoring of self, memory, and reality leading to excessive and false explanations (30). Overall, these observations support the possibility that decreased gray matter volumes in the left hemisphere maybe primarily mediating delusions in patients diagnosed with schizophrenia.

It is possible that delusions exhibited in acute psychosis maybe associated with dopamine hyperactivity in mesolimbic neural pathways (31). The striatum, a mesolimbic structure composed of the caudate, nucleus accumbens, and putamen, receives afferent inputs from various cortical regions, and sends output to the cortex *via* the thalamus (32–34). Striatal dysfunction has been proposed to contribute to psychotic symptoms on the schizophrenia spectrum (35). Our findings of volume loss in the putamen and thalamus support the hypothesis that disruption of these brain regions may be associated with or involved in the generation of delusions in schizophrenia.

In addition, excessive and inappropriate dopamine signaling is thought to render merely coincidental events highly salient (36). The meanings of perceived states of mind may fluctuate based on characteristics of higher levels of brain functionalities. Such cognitive processing may involve prediction errors guiding attention toward events with surprise or uncertainty (37, 38) and engage learning mechanisms (39). Reward prediction errors appear to be principally related to striatal activity while aversive prediction errors are related to insula and habenula activity (40). Activation of the ventral putamen maybe crucial in blocking the prediction-error responses (41). There is also evidence indicating that predictive responses in the putamen are directly related to subjects’ actual behavioral preferences (42). This supports the observation that the putamen was associated with the conscious realization of motor programs and situational conditional reflexes (43). Therefore, volume loss and deficit in the putamen could result in cognitive disruption with the potential to lead to altered attention, learning, and ultimately belief formation and behavior alteration.

The thalamus may play an important role in the development symptoms of schizophrenia (44), relaying multiple signals to and from the cortex (45). Volume loss and deficits in the thalamus may, therefore, result in a functional breakdown in thalamocortical circuitry and lead to the altered cognition and perception often seen as symptoms of schizophrenia (46). Formation of delusions has been hypothesized as associated with the brain’s attempts to integrate the disorganized neural processes experienced by patients diagnosed with schizophrenia. This theory of disorganization in neuronal processes has been supported by observation of widespread volume loss involving fronto-thalamic, insula-claustrum, dorsomedial frontal, and superior temporal cortices observed in the schizophrenia (5–9). Thalamic volume reduction is considered to be a clue to neuropathology underlying schizophrenia and has been speculated as playing a role in disease onset (44). Consistent with this notion, a meta-analysis study (47) on antipsychotic-naive patients of schizophrenia identified volume reductions in caudate nucleus and thalamus, further implying the critical role of the thalamus in the neuropathology of schizophrenia. In this context, our findings may extend the prior evidence to highlight the possible causal association of the thalamus with delusions in patients of schizophrenia.

Several limitations must be noted when interpreting our results. First, the relatively small sample size for the patient groups may indicate less than optimal power of this study. Second, although we are aware of the differences between cortical and subcortical tissue composition based on anatomy, the VBM analysis does not provide the exact composition of gray matter. Future methods that are sensitive to the composition of subcortical tissues will be helpful in clarifying the involvement of different cortical and subcortical structures. Third, medication effects cannot be completely ruled out as some of our participants had been on pharmacotherapy. However, we tried to minimize the confounding medical effect by recruiting first-episode patients who were drug naïve or who had experienced antipsychotic treatment for less than 8 weeks. Fourth, although there was no statistically significant difference on the duration of treatment, SCHnd patients were treated twice as long as the SCHd patients on average, which would be a potentially confounding factor to this study. Finally, all patients reported experiencing psychotic symptoms other than delusions and SCHnd scored higher on the SANS anhedonia subscale, though there were no correlations observed between the SANS anhedonia score and gray matter volume of the left putamen or thalamus. However, prior studies (48–50) observed that anhedonia are associated with both white matter abnormalities (48, 49) and regional gray matter volume (50) in patients with schizophrenia, so the existence of difference on the score of SANS anhedonia between these two patient groups may be another relevant factor in our findings. The two patient groups were matched on all other SAPS symptoms.

In conclusion, our findings suggest that volume loss in the left putamen and thalamus are biologically significant correlates of delusions in schizophrenia. These regions constitute an important component of the cortico–striato–pallido–thalamus circuit, which has been previously implicated in schizophrenia, and reduction in the gray matter volume of these regions has been correlated with severity of symptoms of schizophrenia (51). Future studies are needed to replicate these findings with larger samples, using drug-naïve patients diagnosed with schizophrenia, and their siblings to explore further the underlying neural substrates of delusion.

## Ethics Statement

This study was approved by the Ethics Committee of the Second Xiangya Hospital of Central South University. Written informed signed consent was provided by each participant before being included in the study.

## Author Contributions

XH, WP, XL, AG, SD, ZX, HL, and ZL authored the manuscript. XH, HL, and WP collected the imaging data and clinical information. XH wrote the first draft of the manuscript. All the authors have personally reviewed the manuscript and gave final approval of the version attached.

## Conflict of Interest Statement

The authors declare that the research was conducted in the absence of any commercial or financial relationships that could be construed as a potential conflict of interest. The reviewer JB and handling editor declared their shared affiliation.
